# Interference of miR‐107 with Atg12 is inhibited by HULC to promote metastasis of hepatocellular carcinoma

**DOI:** 10.1002/mco2.25

**Published:** 2020-08-20

**Authors:** Haiming Zhang, Shipeng Li, Haixu Xu, Liying Sun, Zhijun Zhu, Zhi Yao

**Affiliations:** ^1^ Liver Transplantation Center National Clinical Research Center for Digestive Diseases and Beijing Key Laboratory of Tolerance Induction and Organ Protection in Transplantation Beijing Friendship Hospital Capital Medical University Beijing China; ^2^ Department of General Surgery Jiaozuo People's Hospital Xinxiang Medical University Jiaozuo China; ^3^ Department of Immunology Tianjin Key Laboratory of Cellular and Molecular Immunology Tianjin Medical University Tianjin China

**Keywords:** Atg12, autophagy, HCC, HULC, miR‐107

## Abstract

Highly upregulated in liver cancer (HULC) had a significant predictive effect on tumor growth and metastasis of hepatocellular carcinoma (HCC); however, the mechanisms of HULC on HCC still need to be clarified. We attempted to determine the roles of HULC and miR‐107 in autophagy and invasion of HCC. HULC siRNA reduced the level of autophagy. The impact of HULC siRNA on invasion can be reversed by activating autophagy in HCC cell lines. Further studies on HULC and autophagy were conducted. An interacting sequence between HULC and miR‐107, as well as miR‐107 and Atg12, was predicted by software. The relationship of each pair of molecules was confirmed by luciferase reporter assays. The negative impacts of miR‐107 on autophagy and invasion were proved in HCC cell lines. The inhibitor of miR‐107‐promoted invasion can also be reversed by Atg12 siRNA. The changes of miR‐107, Atg12, epithelial‐mesenchymal transition, and autophagy in transplanted tumors of mouse models also confirmed the results in HCC cell lines. Finally, we find that HULC acts as an endogenous sponge, which abolishes the binding of miR‐107 on the Atg12 3′‐UTR and promotes autophagy and metastasis of HCC.

## Graphical abstract

HULC/miR‐107/Atg12 axis seemed to be a relative direct regulation of autophagy. HULC was hypothesized as a ceRNA sequestering miR‐107 and regulates Atg12 indirectly. Then, the role of miR‐107 and Atg12 on HULC‐mediated tumor invasion was confirmed.

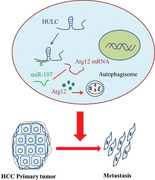

## 1 INTRODUCTION

Highly upregulated in liver cancer (HULC) was proposed as a diagnosis biomarker in hepatocellular carcinoma (HCC).^1^, ^2^, ^3^ HULC promotes tumorigenesis by its impacts on multiple pathways.^2^, ^4^, ^5^, ^6^, ^7^, ^8^ Researchers found in vitro that HULC positively regulates the invasion or migration of gastric cancer cells.^9^ Epithelial‐mesenchymal transition (EMT)^10^ is a key process of the formation of circulating tumor cells^11^ or metastases.^12^, ^13^ It was reported that EMT was reversed after deletion of HULC,^9^ which also supported the role of HULC on tumor dissemination. Autophagy, a mechanism of degradating and recycling of long‐lived proteins and organelles,^14^ was also correlated to EMT.^15^, ^16^, ^17^, ^18^, ^19^ Both of HULC and autophagy were involved in EMT and the progression of tumors. The relationship of HULC and autophagy should be cleared and the role of molecules involved in tumor invasion should be demonstrated.

MiRNAs are noncoding RNAs that plays regulatory roles in multiple diseases and cancers.^20^, ^21^, ^22^ MiRNAs can exert impacts on the growth, EMT, and metastasis of tumors by regulating autophagy.^23^ Some LncRNAs may act as ceRNA to downregulate miRNAs expression and reverse the inhibitory effect of miRNAs.^24^, ^25^ It showed a potential way of HULC acting on downstream molecules to regulate autophagy, EMT, or metastasis. In the current study, the role of autophagy in HULC‐regulated pathway was studied. Finally, we found that HULC promotes HCC invasion by a HULC/miR‐107/Atg12 axis.

## 2 METHODS

### 2.1 Antibodies, RNAs, and viruses

Antibodies against Atg5, Atg7, Atg12, MMP‐2, MMP‐9, LC3, β‐catenin, Claudin‐1, and β‐actin (1:1500, CST, USA) were used in western blot test. LV‐HULC siRNA and GFP‐RFP‐LC3 adenovirus were obtained from the Hanbio Co, Ltd (Shanghai, China). HULC siRNA, Atg12 siRNA, and the mimic and inhibitor of miR‐107 were purchased from the RiboBio Co, Ltd (Guangzhou, China).

### 2.2 Culture and treatment of cells

BEL‐7402 and SMMC‐7721, two human HCC cell lines, were purchased from ATCC (Manassas, VA, USA), which were grown in RMPI1640 medium (Biological Industries, Kibbutz Beit‐Haemek, Israel). The above chosen HCC cell lines were supplemented with 10% FBS and 100 µg/mL each of penicillin and streptomycin (Gibco, New York, NY, USA). Autophagy was activated by Rapamycin (50 nM, Sigma Aldrich, St. Louis, MO, USA) and inhibited by 3‐methyladenine (3‐MA; 75µM, Selleck Chemicals, Houston, TX, USA). Cells were transfected by Lipofectamine 3000 (Invitrogen, Carlsbad, CA, USA).

### 2.3 Animal experiments

All animal experiments were conducted after the approval of the ethics committee of Beijing Friendship Hospital. The transfected SMMC‐7721 cells were injected subcutaneously into the flanks of each 4‐week‐old Balb/c athymic nude mice. After 5 weeks of tumor growth, mice were euthanized, and necropsies were performed. SMMC‐7721 cells were injected subcutaneously into the left liver lobes of each nude mouse. Five weeks after SMMC‐7721 cells implantation, the mice were sacrificed to examine via hematoxylin and eosin (H&E) staining.

### 2.4 Dual luciferase reporter assay

HCC cells were co‐transfected with either miR‐NC/miR‐107 mimic, luciferase reporter comprising 3′‐UTR of Atg12, or wild/mutant Atg12 fragment. Dual luciferase reporter assay was detected according to the manufacturer's protocol.

### 2.5 Cell EMT model

SMMC‐7721 and BEL‐7402 cells were seeded in DMEM containing 1% FBS with or without TGF‐β1 (10 ng/mL, PeproTech, USA), and then culture was continued for an additional 5 days.

### 2.6 RNA extraction and quantitative real‐time reverse transcription polymerase chain reaction analysis

Total cellular RNA was extracted and real‐time reverse transcription polymerase chain reaction (RT‐PCR) were performed according to the manufacturer's instructions.

### 2.7 Western blot test, invasion/migration assay, immunohistochemical staining, and immunofluorescence staining

Western blot test, invasion/migration assay, immunohistochemical (IHC) staining, and immunofluorescence (IF) staining were conducted as reported previously.^4^, ^26^

### 2.8 Statistical analysis

A SPSS 22.0 statistical software package was used for statistical analysis. The significance of differences was estimated by *t*‐test, one‐way analysis of variance, *χ* ^2^ test, or Mann‐Whitney *U* test. All tests performed were two sided and the criterion for statistical significance was taken as *P* < .05.

## 3 RESULTS

### 3.1 HULC promotes invasion/migration by activating autophagy

**FIGURE 1:**
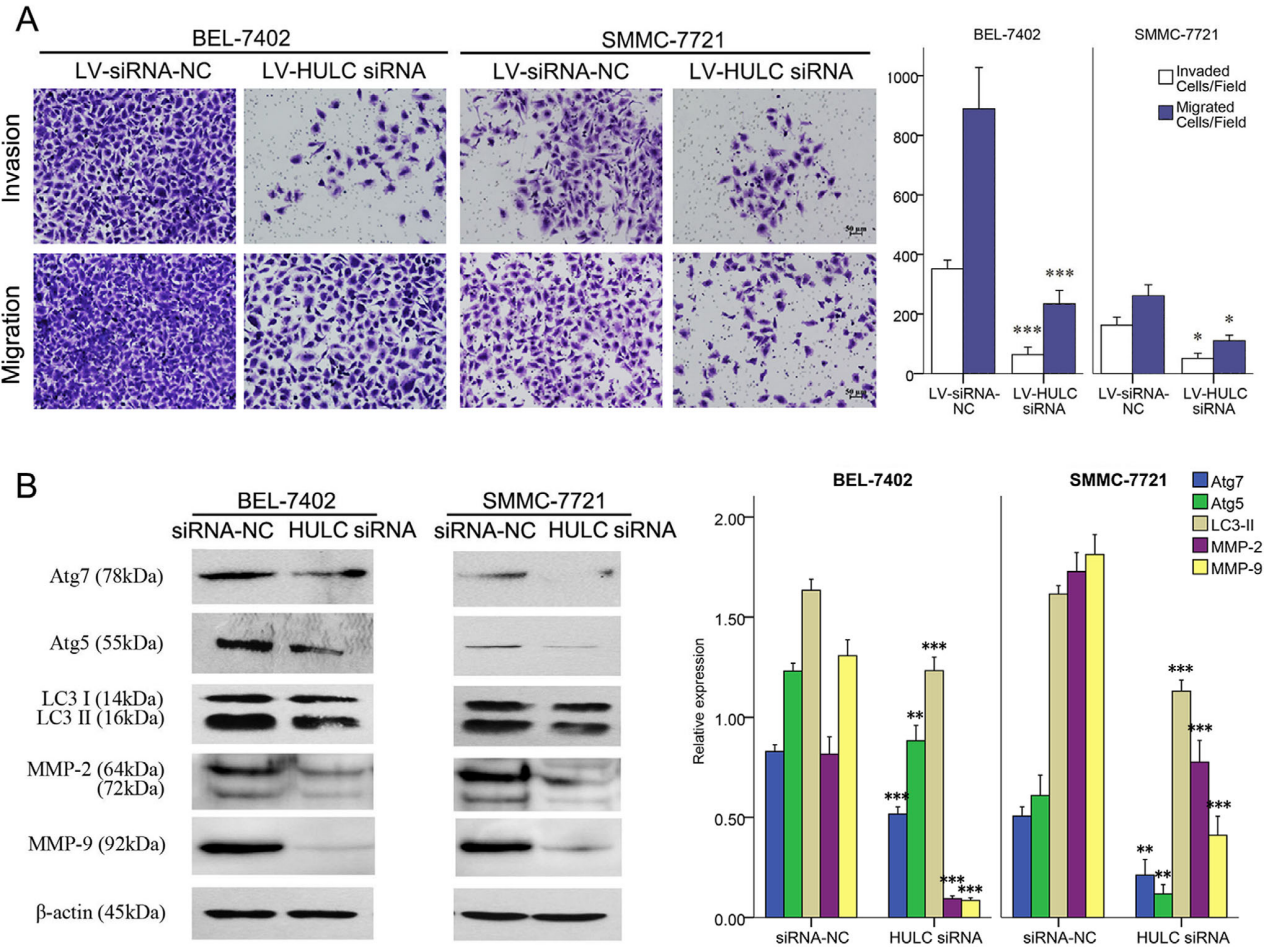
The impact of HULC on metastasis of BEL‐7402 and SMMC‐7721 cells. A, Invasion and migration of cancer cells in transwell assays after transfected by LV‐HULC siRNA or LV‐siRNA NC in SMMC‐7721 and BEL‐7402 cell lines. B, expressions of MMP‐2, MMP‐9, and Atg proteins quantified by western blots analysis in SMMC‐7721 and BEL‐7402 cell lines after a treatment of siRNA NC or HULC siRNA. ^*^P < .05, ^**^ *P* < .01, and ^***^ *P* < .001 (compared with the first group)

According to our previous investigation,^4^ high levels of HULC were found in BEL‐7402 and SMMC‐7721 in comparison with L02. HULC siRNA was selected to investigate its function by blocking its interaction with other molecules, because over expressing HULC was supposed to have a ceiling effect in this context. In the migration and invasion assays, fewer cells migrated or invaded into the lower surface of filter (Figure 1A) and it showed a compromised ability of invasion/migration in HCC cells harboring HULC siRNA. Low expressions of MMP‐2 and MMP‐9 in HULC siRNA group were also found by western blot analysis (Figure 1B). Both of these two enzymes can mediate basement membrane breakdown and invasion. These results showed a negative role of HULC siRNA on migration/invasion of liver cancer cells.

**FIGURE 2:**
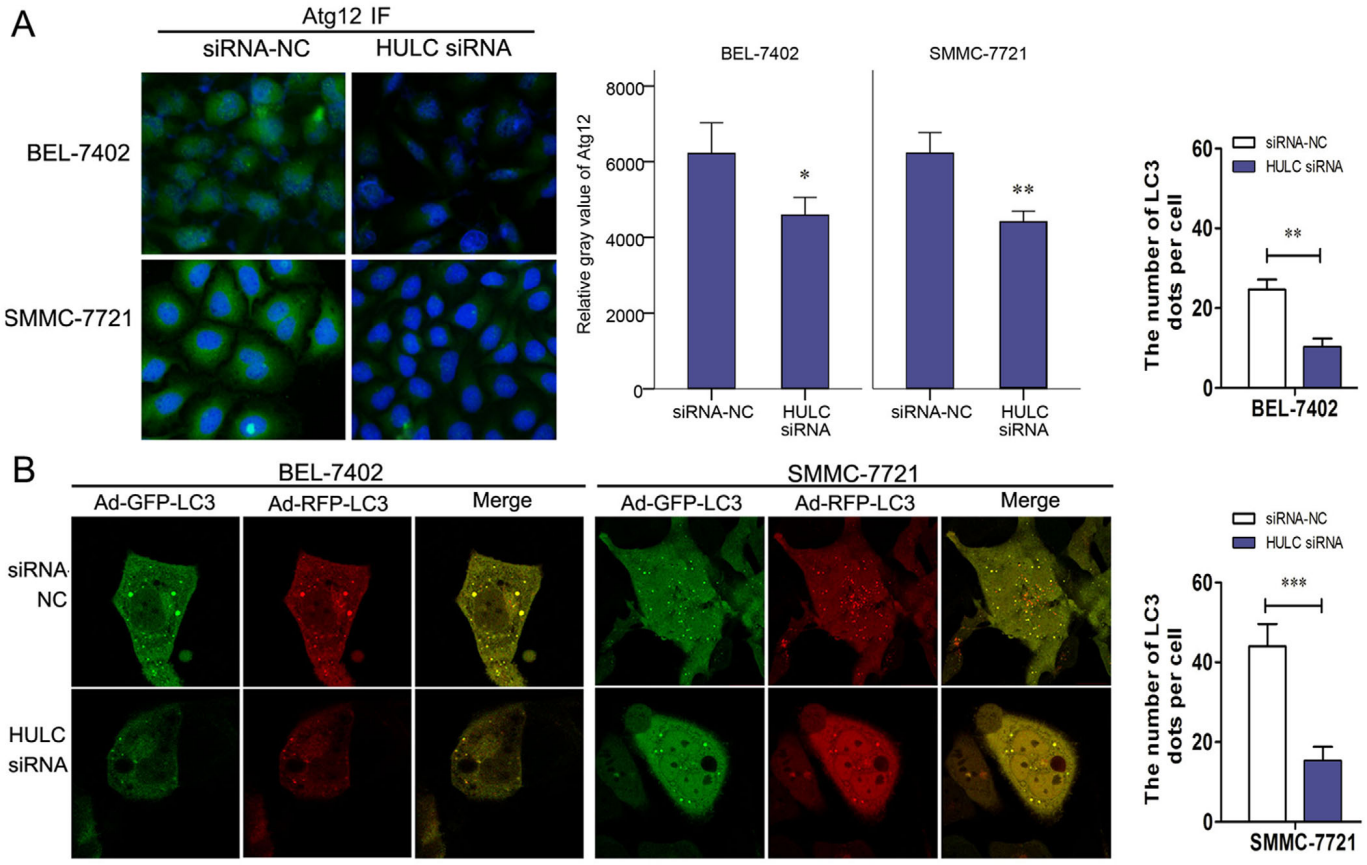
Role of HULC on invasion and autophagy in BEL‐7402 and SMMC‐7721 cells. A, Immunofluorescence (IF) stain for Atg12 (green) in siRNA NC‐ or HULC siRNA‐transfected BEL‐7402 and SMMC‐7721 cells. B, After autophagy was visualized by a mRFP‐GFP‐LC3 adenoviral vector, images under a confocal laser scanning microscope showed the numbers of LC3 fluorescent puncta in siRNA NC or HULC siRNA (100 nmol/L) transfected cells (SMMC‐7721 and BEL‐7402). ^*^ *P* < .05, ^**^ *P* < .01, and ^***^ *P* < .001 (compared with the first group)

In HCC cells treated with HULC siRNA, the levels of LC3‐II, Atg5, and Atg7 decreased significantly, as well as the level of Atg12 shown by IF staining (Figures 1B and 2A), suggesting the reduction of Atg12 conjugation system (Atg12/Atg7/Atg5) and autophagy. Then, autophagy in HCC cells was visualized using an mRFP‐GFP‐LC3 adenovirus vector and observed under a confocal laser scanning microscope. Comparing with the HULC siRNA NC, there were significant decreases in the number of green, red, or merged fluorescent puncta after HULC siRNA administration (Figure 2B). It confirmed a positive role of HULC on autophagy.

**FIGURE 3:**
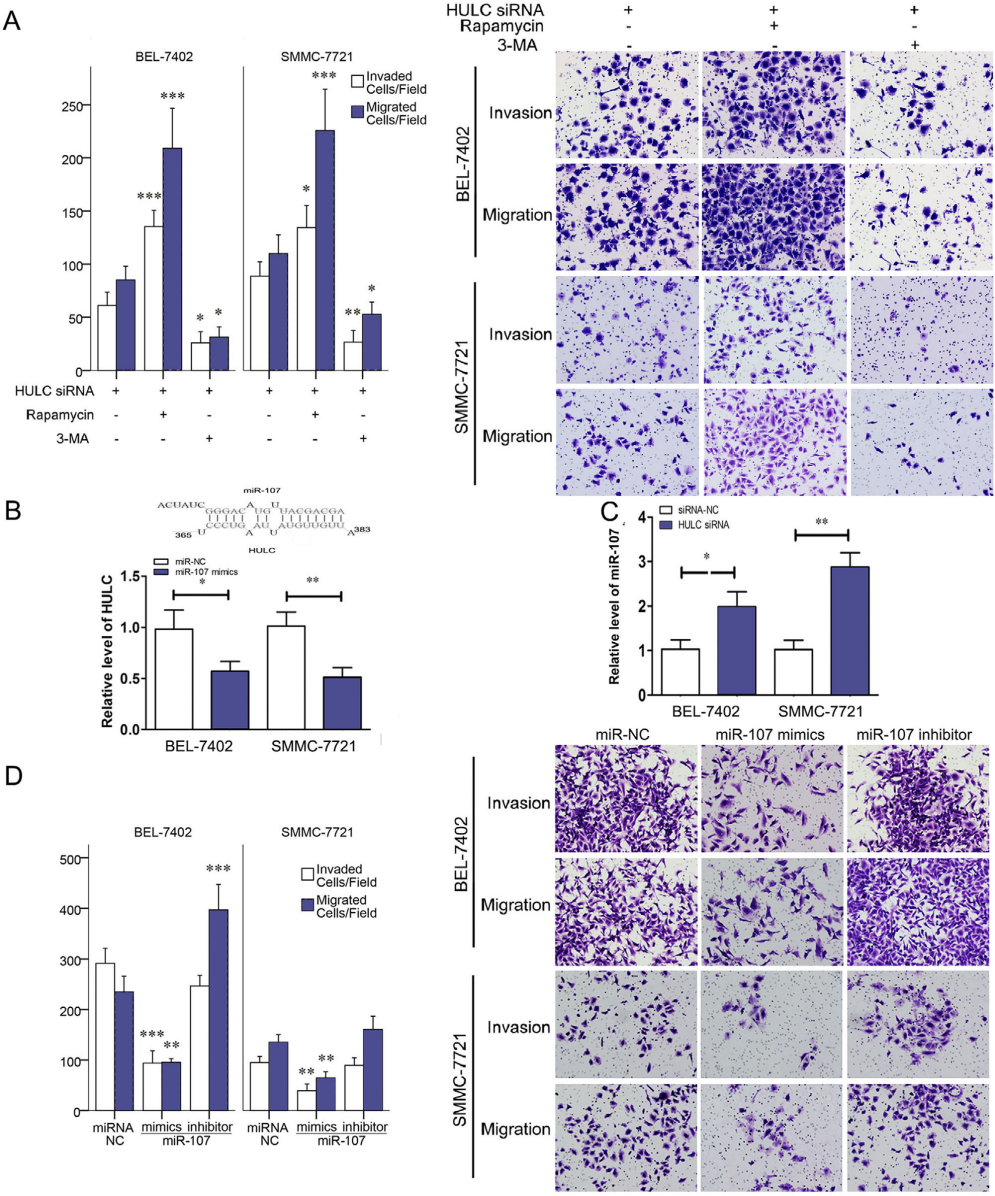
HULC‐promoted invasion via miR‐107 and autophagy. A, Invasion and migration of SMMC‐7721 and BEL‐7402 cell lines were showed in transwell assays after HULC siRNA, HULC siRNA+Rapamycin, or HULC siRNA+3‐MA treatment. B, The predicted interaction between HULC and miR‐107 through complementary base‐pairs and qRT‐PCR results of HULC in BEL‐7402 and SMMC‐7721 cells transfected with miR‐107 NC or miR‐107 mimic. C, qRT‐PCR results of miR‐107 in BEL‐7402 and SMMC‐7721 cells transfected with siRNA‐NC or HULC siRNA. D, Invasion and migration of SMMC‐7721 and BEL‐7402 cell lines in transwell assays, after miR‐107 NC, miR‐107 mimic, or miR‐107 inhibitor treatment. ^*^P < .05, ^**^ *P* < .01, and ^***^ *P* < .001 (compared with the first group)

After the roles of HULC in invasion/migration and autophagy were found, the impact of autophagy on HULC‐regulated invasion/migration was studied. In transwell assays, the invasion/migration of HULC siRNA‐treated cells was increased after Rapamycin administration and decreased after 3‐MA administration (Figure 3A). Thus, autophagy has a significant impact on invasion/migration as HULC. Autophagy promoted by HULC can serve as one of the pathways in HULC‐regulated invasion/migration.

### 3.2 HULC promotes invasion/migration by inhibiting miR‐107

To clarify the underlying mechanism by which HULC has impact on autophagy, a direct target of HULC was predicted using a TargetScan6.2 bioinformatics algorithm and a miRanda software. In alignment prediction, HULC was aligned with a sequence of miR‐107 (Figure 3B). Then, further experiments were conducted in cell lines. Compared with cells treated with siRNA‐NC, HULC siRNA‐transfected HCC cells have a significant increase in level of miR‐107 quantified by Quantitative Real‐time Polymerase Chain Reaction (qRT‐PCR), and a reduction of HULC expression was found after miR‐107 mimic introduction (Figures 3B and 3C). All these results confirmed negative regulations between HULC and miR‐107 by binding with each other.

**FIGURE 4:**
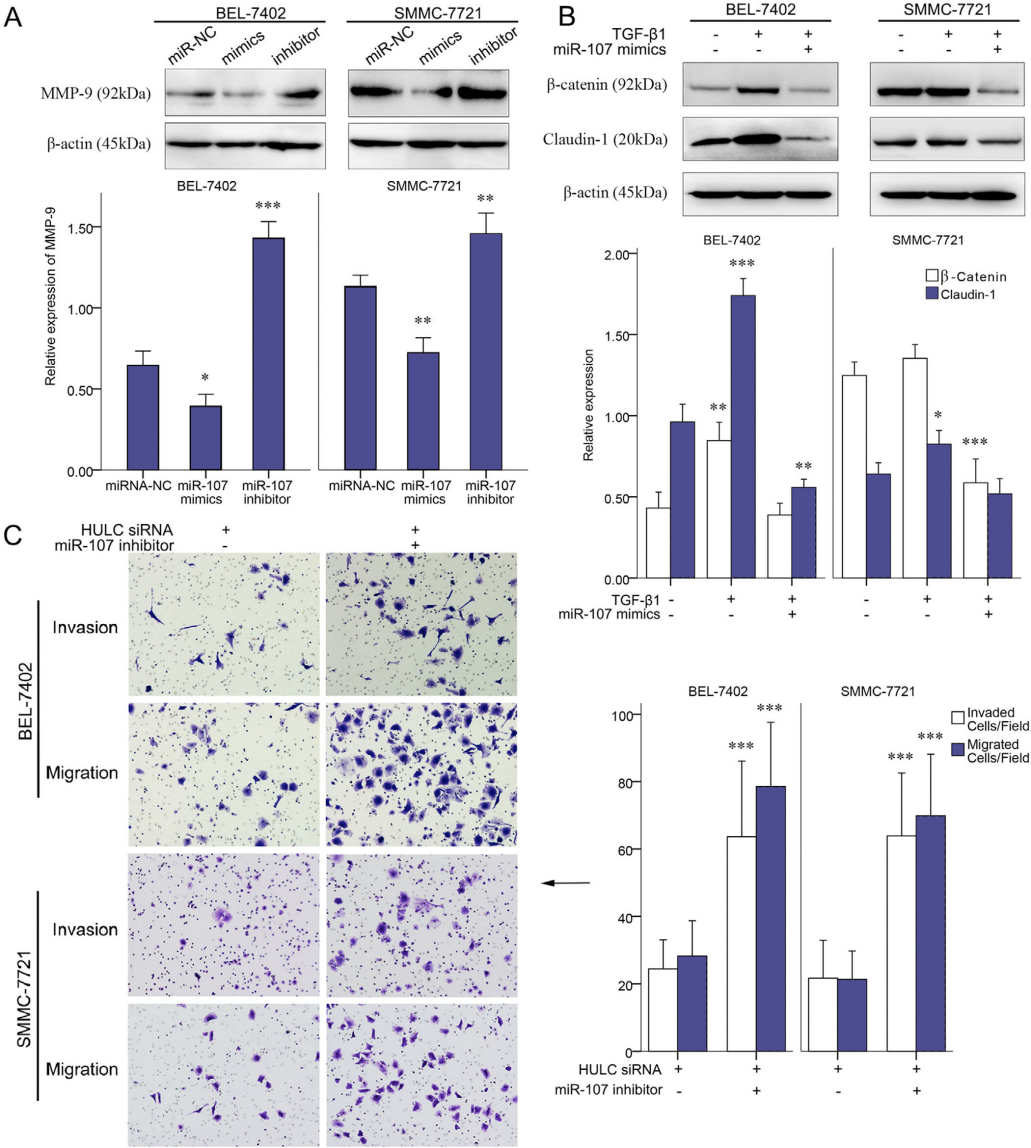
Role of miR‐107 on invasion of BEL‐7402 and SMMC‐7721 cells. A, Expressions of MMP‐9 quantified by western blots analysis in BEL‐7402 and SMMC‐7721 cells treated with miR‐107 NC, miR‐107 mimic, or miR‐107 inhibitor. B, Expressions of β‐catenin and claudin‐1 quantified by western blots analysis in the control, TGF‐β1, and TGF‐β1+miR‐107 mimic groups of BEL‐7402 and SMMC‐7721 cells. C, Invasion and migration of SMMC‐7721 and BEL‐7402 cell lines in transwell assays, after HULC siRNA or HULC siRNA+miR‐107 inhibitor treatment. ^*^P < .05, ^**^ *P* < .01, and ^***^ *P* < .001 (compared with the first group)

The impact of miR‐107 on cancer cell invasion/migration was studied by a transwell assay with HCC cells. The numbers of invading/migrating cells were decreased in miR‐107 mimic group and increased in miR‐107 inhibitor group (Figure 3D). Western blots analyses were also made to quantify proteins in miR‐107 mimic‐ or inhibitor‐treated BEL‐7402 and SMMC‐7721 cells. MMP‐9 was found to be decreased in miR‐107 mimic group and increased in miR‐107 inhibitor group (Figure 4A). β‐Catenin and claudin‐1, two hallmarks of EMT, were compared among control, TGF‐β, and TGF‐β + miR‐107 mimic groups. Significant reductions of β‐catenin and claudin‐1 were found after a miR‐107 mimic was added to the TGF‐β‐incubated cells (Figure 4B). These results demonstrated that miR‐107 inhibits the invasion, migration, and EMT among liver cancer cells.

After knocking HULC down by HULC siRNA, invasion/migration of HCC cells in transwell assays was promoted by a miR‐107 inhibitor (Figure 4C). Thus inhibiting miR‐107 has a positive role in the invasion/migration. Inhibiting miR‐107 by HULC can serve as a pathway in HULC‐regulated invasion/migration.

### 3.3 miR‐107 regulates the expression of Atg12

**FIGURE 5:**
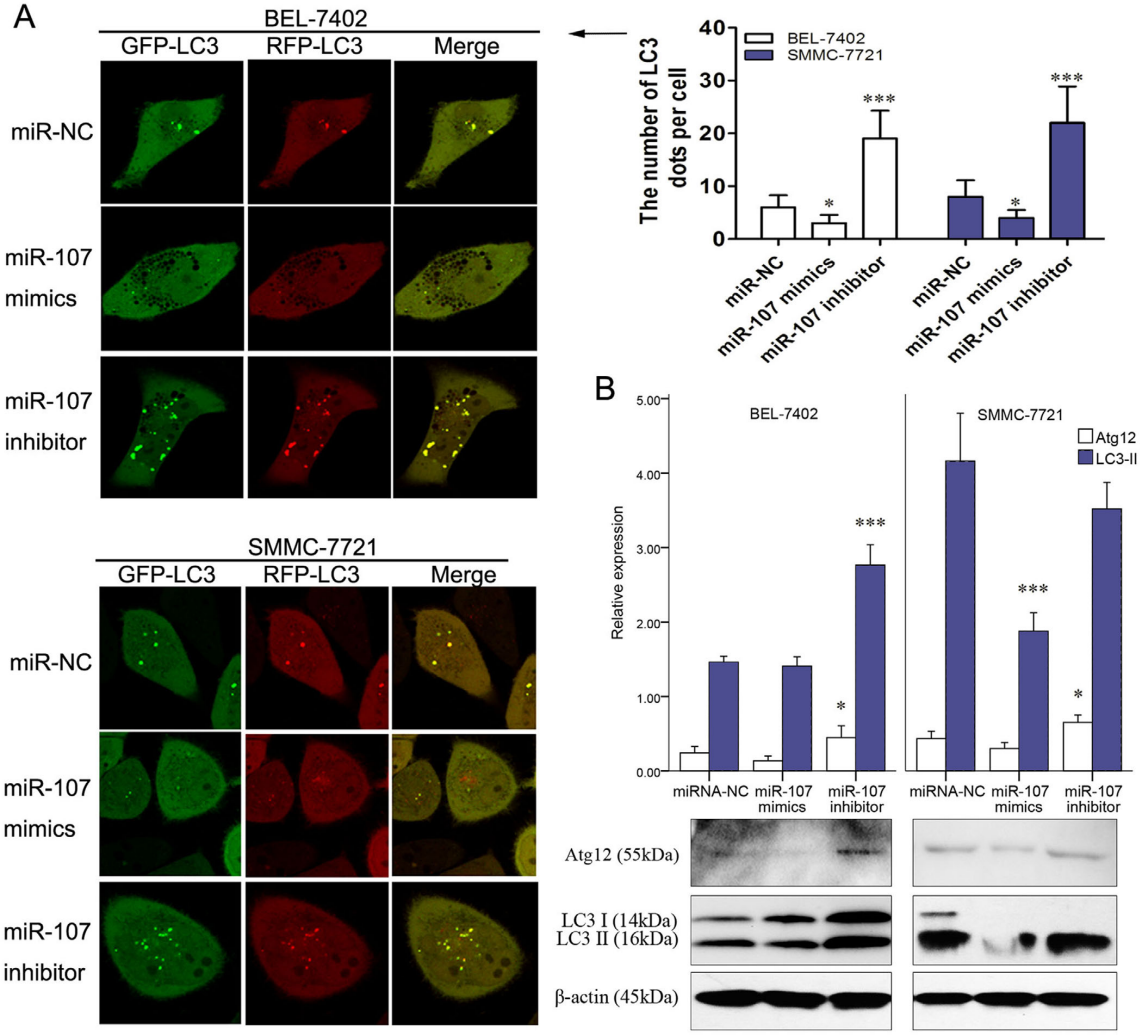
Autophagy was inhibited by miR‐107. A, After autophagy was visualized by a mRFP‐GFP‐LC3 adenoviral vector, images under a confocal laser scanning microscope showed the numbers of LC3 fluorescent puncta in miR‐107 NC, miR‐107 mimic, or miR‐107 inhibitor‐treated cells (SMMC‐7721 and BEL‐7402). B, Expressions of Atg12, LC3‐I, and LC3‐II quantified by western blots analysis in miR‐107 NC, miR‐107 mimic, or miR‐107 inhibitor groups. ^*^ *P* < .05, ^**^ *P* < .01, and ^***^ *P* < .001 (compared with the first group)

Because autophagy and miR‐107 both had roles in HULC‐regulated pathway, the impact of miR‐107 on autophagy was studied. After treating with miR‐107 mimic or inhibitor, mRFP‐GFP‐LC3 was overexpressed by mRFP‐GFP adenoviral vector in HCC cells. The fluorescent puncta of autophagosomes were observed by a confocal laser scanning microscope. Comparing with the miR‐NC group, the numbers of green, red, and merged fluorescent puncta decreased in miR‐107 mimic group and increased in miR‐107 inhibitor group (Figure 5A). Similarly, Atg12 and LC3‐II were found to be decreased in miR‐107 group and increased in miR‐107 inhibitor group (Figure 5B). These results confirmed the inhibitive role of miR‐107 on autophagy.

**FIGURE 6:**
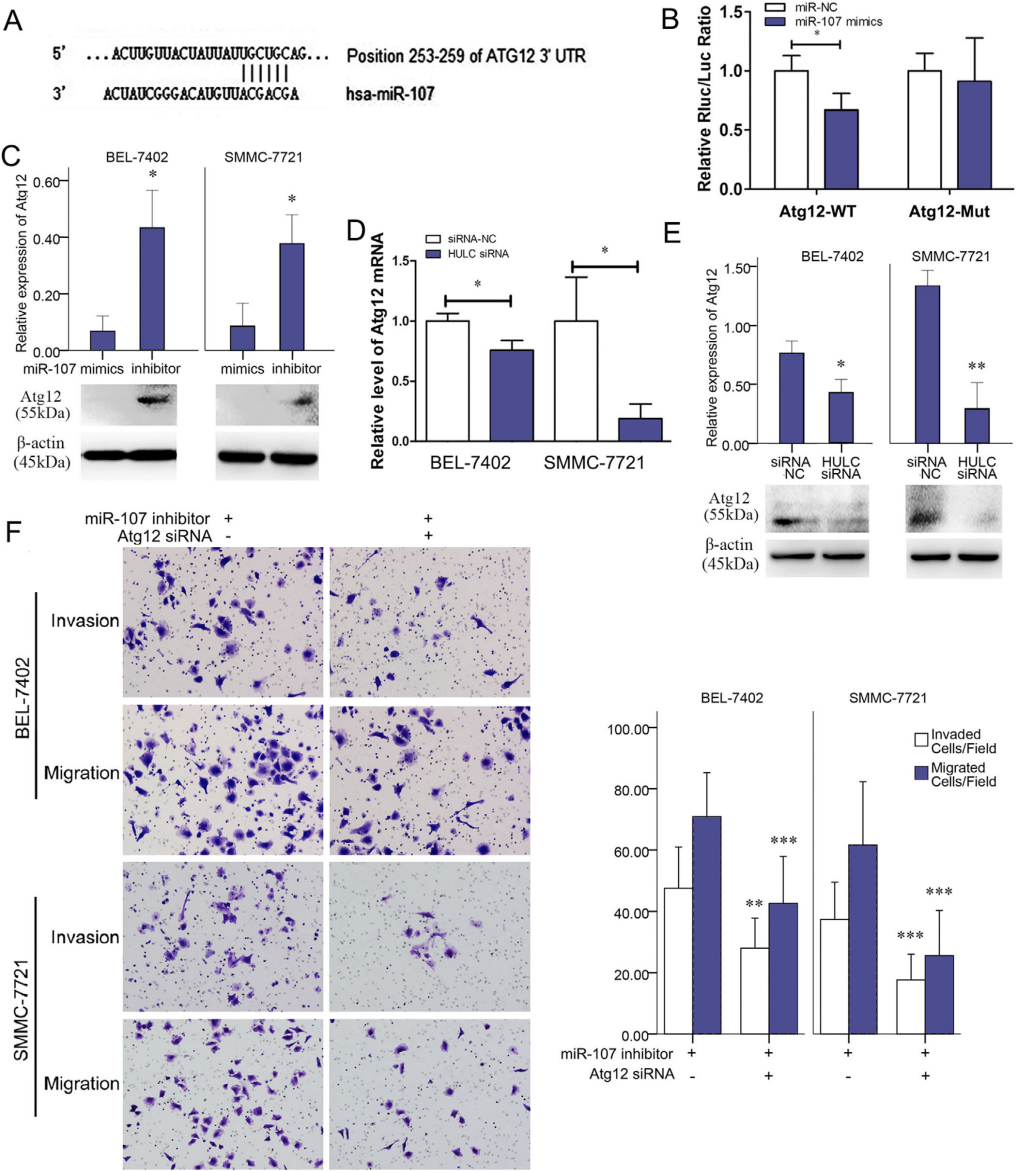
miR‐107‐inhibited autophagy through interfering with Atg12 mRNA. A, The predicted interaction between miR‐107 and Atg12 mRNA through complementary base‐pairs. B, In SMMC‐7721 and BEL‐7402 cells, luciferase activities in SMMC‐7721 cells transfected with miR‐107 NC or miR‐107 mimic, together with luciferase reporter plasmid harboring wild‐type sequence of Atg12 mRNA or mutated sequence of Atg12 mRNA. C, Expressions of Atg12 quantified by western blots analysis after miR‐107 mimic or miR‐107 inhibitor treatment. D, Expressions of Atg12 mRNA quantified by qRT‐PCR in siRNA NC and HULC siRNA groups. E, Expressions of Atg12 quantified by western blots analysis in siRNA NC and HULC siRNA groups. F, Invasion and migration of cancer cells in miR‐107 inhibitor or miR‐107 inhibitor+Atg12 siRNA group. ^*^ *P* < .05, ^**^ *P* < .01, and ^***^ *P* < .001 (compared with the first group)

A conjugating sequence of miR‐107 to the Atg12 3′‐UTR (position 253–259) was also found by the abovementioned software (Figure 6A). In Figure 6B, miR‐107 mimic significantly decreased luciferase activity in cells treated by the reporter plasmid with wild‐type targeting sequence of Atg12 mRNA, but not with reporter plasmid with mutated Atg12 mRNA. To further confirm the role of miR‐107 in the expression of Atg12, we detected the Atg12 expression in HCC cells transfected with miR‐107 mimic or miR‐107 inhibitor. A reduced expression of Atg12 in miR‐107 mimic group was found by western bot analysis, as compared with miR‐107 inhibitor group (Figure 6C).

### 3.4 Atg12 is critical in HULC/miR‐107‐regulated invasion/migration

Similar to the Atg12 expression found in miR‐107 mimic group (Figure 6C), Atg12 were apparently downregulated in HULC siRNA‐transfected cells (Figures 6D and 6E). These results confirmed the regulation of miR‐107 and HULC on Atg12. HULC may act as an endogenous sponge, which abolishes the binding of miR‐107 on the Atg12 3′‐UTR. In transwell assay, invasions/migrations of miR‐107 inhibitor‐treated HCC cells were suppressed by co‐treatment of Atg12 siRNA (Figure 6F). Thus, Atg12 is also critical in the HULC/miR‐107‐regulated invasion/migration.

### 3.5 MiR‐107, Atg12, EMT, and autophagy in transplanted tumors of mouse models

**FIGURE 7:**
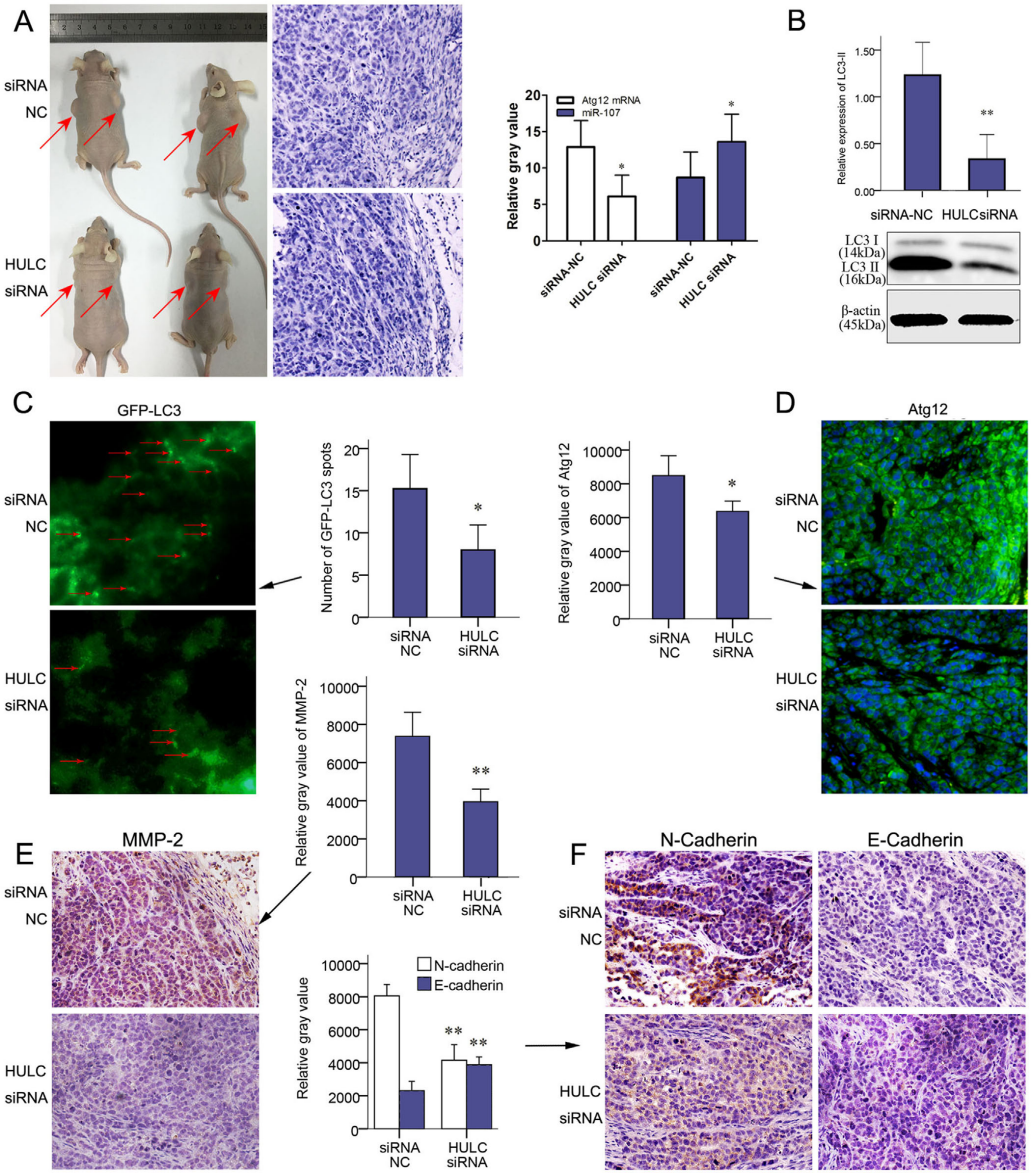
HULC‐promoted invasion of subcutaneous planted tumors. After transfecting with a LV‐HULC siRNA or LV‐siRNA‐NC, SMMC‐7721 cells were implanted (n = 8) into 5‐week‐old nude mice subcutaneously. A, Tumors in situ (red arrows), H&E staining of para‐tumor tissues, and qRT‐PCR results of miR‐107 and Atg12 mRNA in xenograft tumors in siRNA NC and HULC siRNA groups. B, The expression of LC3 quantified by western blots analysis in siRNA NC and HULC siRNA groups. C, At the end of the 5th week, 25 µL mRFP‐GFP LC3 adenoviral virus (1×10^10^ PFU/mL) was injected into the xenograft tumors of both groups to detect autophagy. Forty‐eight hours later, tumors were harvested and studied. Green puncta of autophagosomes were captured by fluorescence microscope in frozen sections of tumors. D, Expressions of Atg12 were analyzed by IF staining on paraffin section. (E) Expressions of MMP2 and (F) N‐Cadherin and E‐cadherin were showed by IHC staining in HULC siRNA and siRNA‐NC groups (magnification, 200×; scale bars = 50 µm). ^*^ *P* < .05 and ^**^ *P* < .01 (compared with the first group)

After transfecting with a LV‐HULC siRNA, SMMC‐7721 cells were implanted into nude mice subcutaneously (Figure 7A). In sections of tissues around the tumor, less invaded tumor cells were found in HULC siRNA group (Figure 7A). In HULC siRNA group, a relative low level of Atg12 mRNA and high level of miR107 were found by qRT‐PCR (Figure 7A). Autophagy activation represented as LC‐II in western blots analysis also decreased in HULC siRNA group (Figure 7B). mRFP‐GFP LC3 adenoviral vectors were injected before tumor harvesting and fluorescent puncta of autophagosomes on the frozen sections were compared between groups. The numbers of fluorescent puncta decreased in HULC siRNA group, compared with siRNA NC group (Figure 7C). Atg12 visualized by IF staining also decreased in HULC siRNA group (Figure 7D). Compared to siRNA‐NC groups, the expression of MMP‐2 and N‐cadherin decreased in HULC siRNA group, and the expression of E‐cadherin increased, which suggested a compromised ability of invasion or EMT (Figures 7E and 7F).

**FIGURE 8:**
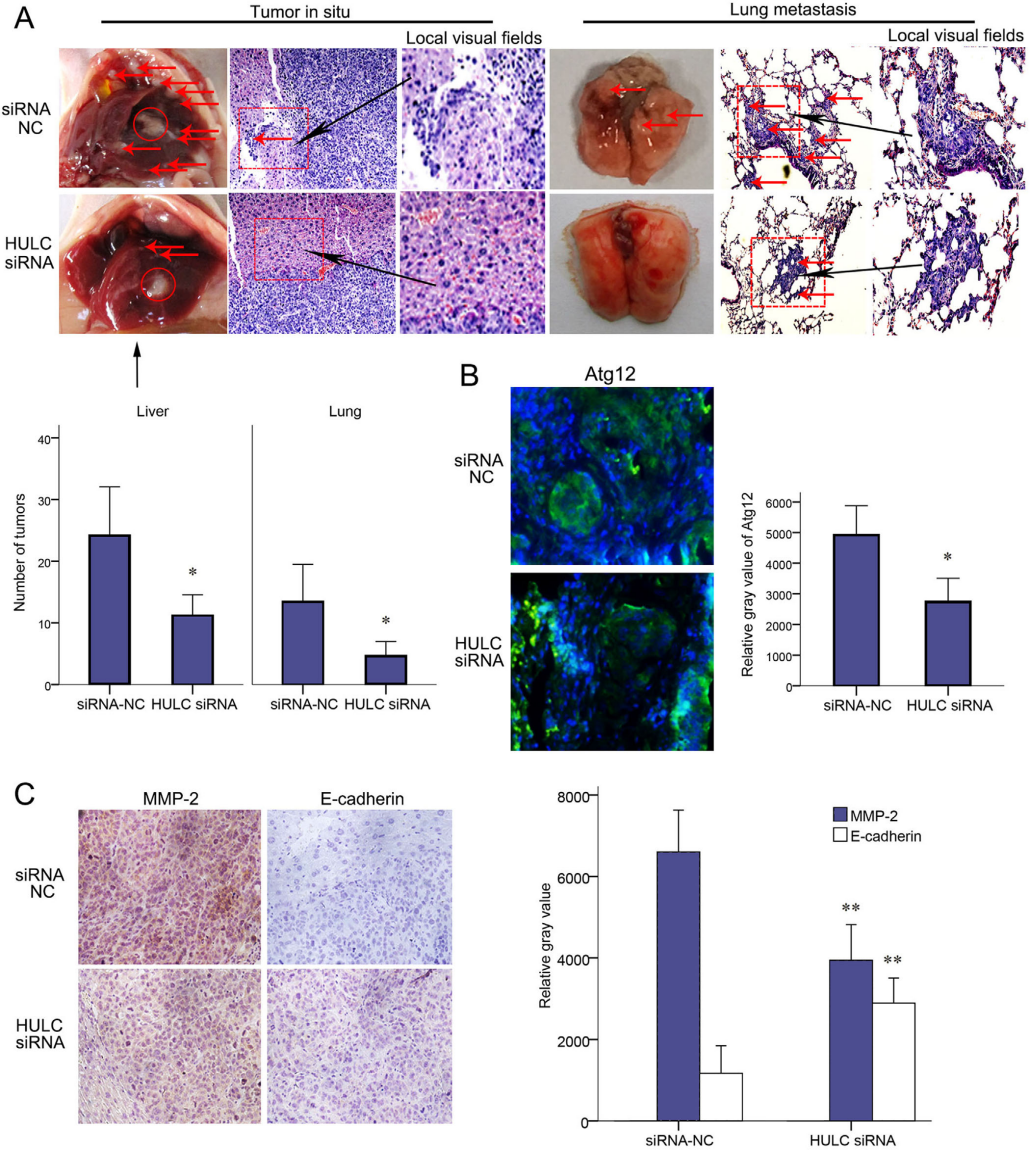
HULC‐promoted EMT and metastasis of intrahepatic planted tumors. SMMC‐7721 cells transfected with LV‐HULC siRNA or siRNA‐NC were injected into the livers of nude mice. A, Five weeks after implantation, tumor nodules (red arrows) were observed on the surface of liver, diaphragm, and lung. Histological presentations of tumors were showed by H&E staining of paraffin sections (magnification, 200×; scale bars = 50 µm). B, IF staining for Atg12 on paraffin sections of pulmonary metastases. C, IHC staining for MMP‐2 and E‐cadherin on paraffin sections (magnification, 200×; scale bars = 50 µm). ^*^ *P* < .05 and ^**^ *P* < .01 (compared with the first group)

SMMC‐7721 cells with HULC siRNA or siRNA‐NC were in situ injected into mouse livers. More tumor nodules within and around liver were found in siRNA‐NC group (Figure 8A). There were also more pulmonary metastatic lesions in siRNA‐NC group than HULC siRNA group (Figure 8A). On the sections of pulmonary metastatic tumors, IF stain of Atg12 decreased in HULC siRNA group (Figure 8B). IHC staining of intrahepatic tumor sections showed a decreased MMP‐2 and an increased E‐cadherin in HULC siRNA group (Figure 8C).

## 4 DISCUSSION

The level of HULC was correlated with clinical outcome in many cancers.^9^, ^27^, ^28^ Hämmerle et al found HULC higher in low‐grade and low‐stage HCC.^29^ Xie et al reported that higher HULC expression was associated with Tumor‐Lymph Node‐Metastasis (TNM) stages of HCC.^3^, ^30^ Li et al reported the predictive role of HULC on tumor growth and metastasis of HCC.^30^ However, the mechanisms of HULC in HCC still need to be cleared.

It was reported that autophagic flux can be induced by environmental stresses.^32^ Further studies found autophagy promoted not only the survival of dormant tumor cells and disseminating them to circulation but also directly regulated the EMT and also worked as a responsor in the metastatic cascade.^15^, ^16^ Autophagy may be involved in HULC‐regulated pathway. To clear the function of autophagy in HULC‐regulated liver cancer invasion, autophagy was activated by Rapamycin after HULC was knocked down. Then, the negative impact of HULC siRNA on invasion/migration of liver cancer cells was reversed by activating autophagy (Figure 3A). Thus autophagy is important for HULC to promote invasion. Molecular mechanisms between HULC and autophagy should be cleared.

Atg proteins, such as Atg12 and Atg5, can bind to the autophagic membranes, as key regulators of the autophagic process.^33^ Unlike P62, the expression of Atg proteins may directly impact the autophagy,^34^, ^35^ which was also correlated to differentiation and metastasis of gastric cancer.^36^ The relation between HULC and expression of these proteins was explored in this study. Instead of direct conjugating sequences between HULC and Atg proteins, a sequence of HULC acting on miR107 and a sequence of miR107 acting on Atg12 were found by a prediction software (Figures 3B and 6A). LncRNA might function as molecular sponges for miRNAs.^37^, ^38^, ^39^ According to the findings in conjugating sequences, HULC was hypothesized as a ceRNA sequestering miR‐107 and regulates Atg12 indirectly. Then, the role of miR‐107 and Atg 12 in HULC‐mediated tumor invasion was confirmed in following examinations.

The functions of miR‐107 on growth and invasiveness are controversial in different types of carcinomas.^40^, ^41^, ^42^ The target molecules of miR‐107 and pathways involved have not been fully understood. The affinity between HULC and miR‐107 is higher than the affinity between miR‐107 and Atg12 mRNA. However, the affinities between miR‐107 and some of the other conjugating molecules may be stronger. Thus, miR‐107 still has a potential to take part in other processes after its combination with HULC. HULC may remove part of its functions and reserve other functions, such as promoting tumor progression. The cross‐talks between molecules conjugating with HULC or miR‐107 should also be further investigated. The HULC/miR‐107/Atg12 axis serves as a critical pathway to promote autophagy and finally results in metastasis of high HULC expressing HCCs.

## AUTHOR CONTRIBUTIONS

ZHM, LSP, and XHX performed the majority of experiments and analyzed the data. LSP and XHX performed the molecular investigations. LSP and XHX participated in treatment of animals. SLY, ZZJ, and YZ designed the research. ZHM wrote the paper.

## ETHICS APPROVAL

The approval of animal experiments was acquired from the ethics committee of Beijing Friendship Hospital.

## AVAILABILITY OF DATA AND MATERIALS

All data generated or analyzed during this study are included in this published article.

## CONFLICT OF INTEREST

The authors declared no conflict of interest.

## Supporting information

Supporting Information
