# Correction to: Interference of miR‐107 with Atg12 is inhibited by HULC to promote metastasis of hepatocellular carcinoma

**DOI:** 10.1002/mco2.215

**Published:** 2023-01-31

Haiming Zhang, Shipeng Li, Haixu Xu, Liying Sun, Zhijun Zhu, Zhi Yao

First published: 20 August 2020, https://doi.org/10.1002/mco2.25

In the process of checking the raw data^1^, the authors noticed several inadvertent mistakes occurring in Figure 1A, Figure 3D, Figure 4C and Figure 6F during the preparation of these panels. The correct results should be as shown below. The authors apologize for these oversights and declare that these corrections do not affect the description, interpretation, or conclusions detailed in the original manuscript.

**FIGURE 1:**
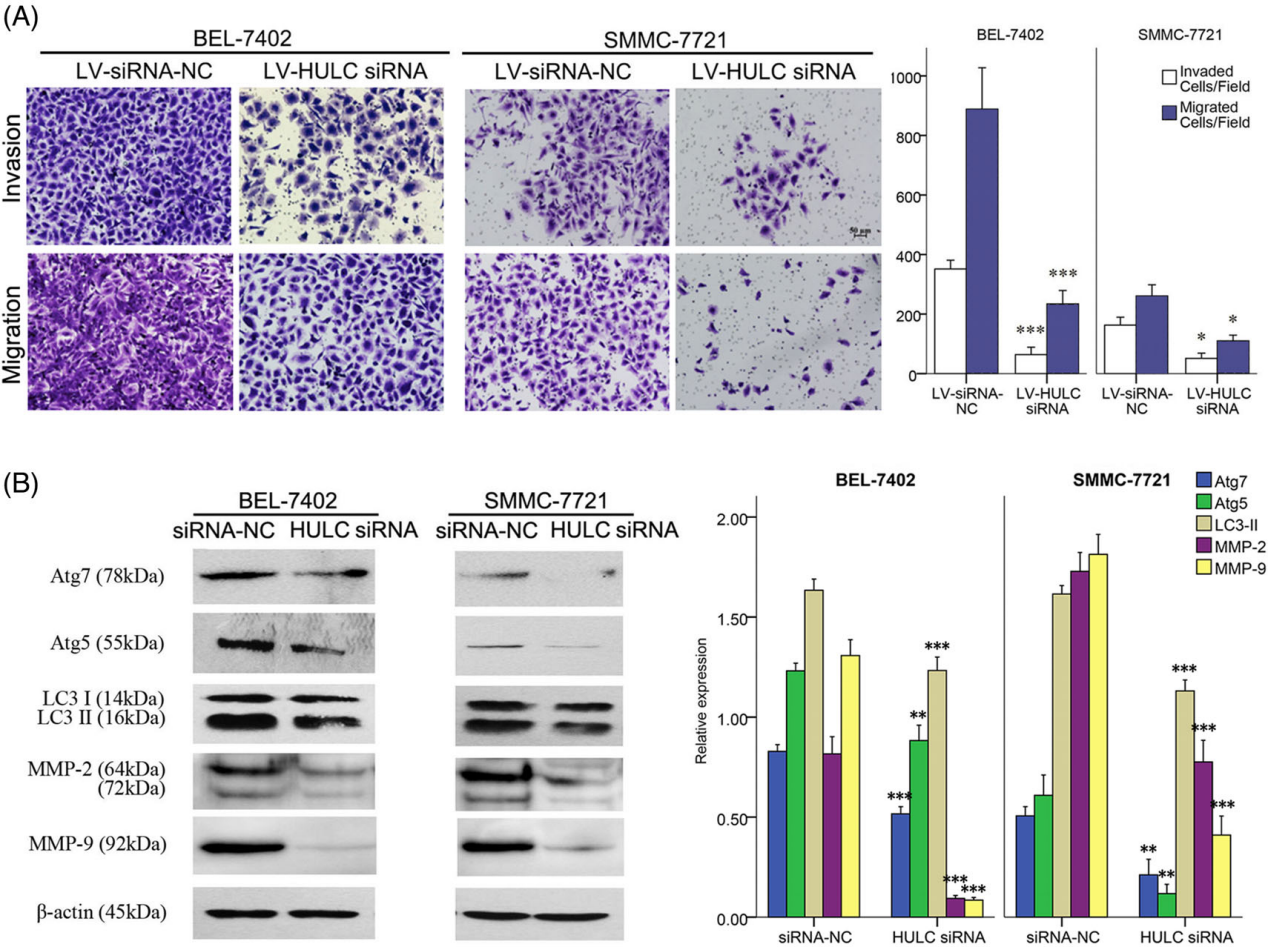
The impact of HULC on metastasis of BEL‐7402 and SMMC‐7721 cells. A, Invasion and migration of cancer cells in transwell assays after transfected by LV‐HULC siRNA or LV‐siRNA NC in SMMC‐7721 and BEL‐7402 cell lines. B, expressions of MMP‐2, MMP‐9, and Atg proteins quantified by western blots analysis in SMMC‐7721 and BEL‐7402 cell lines after a treatment of siRNA NC or HULC siRNA. *P < .05, **P < .01, and ***P < .001 (compared with the first group)

**FIGURE 3:**
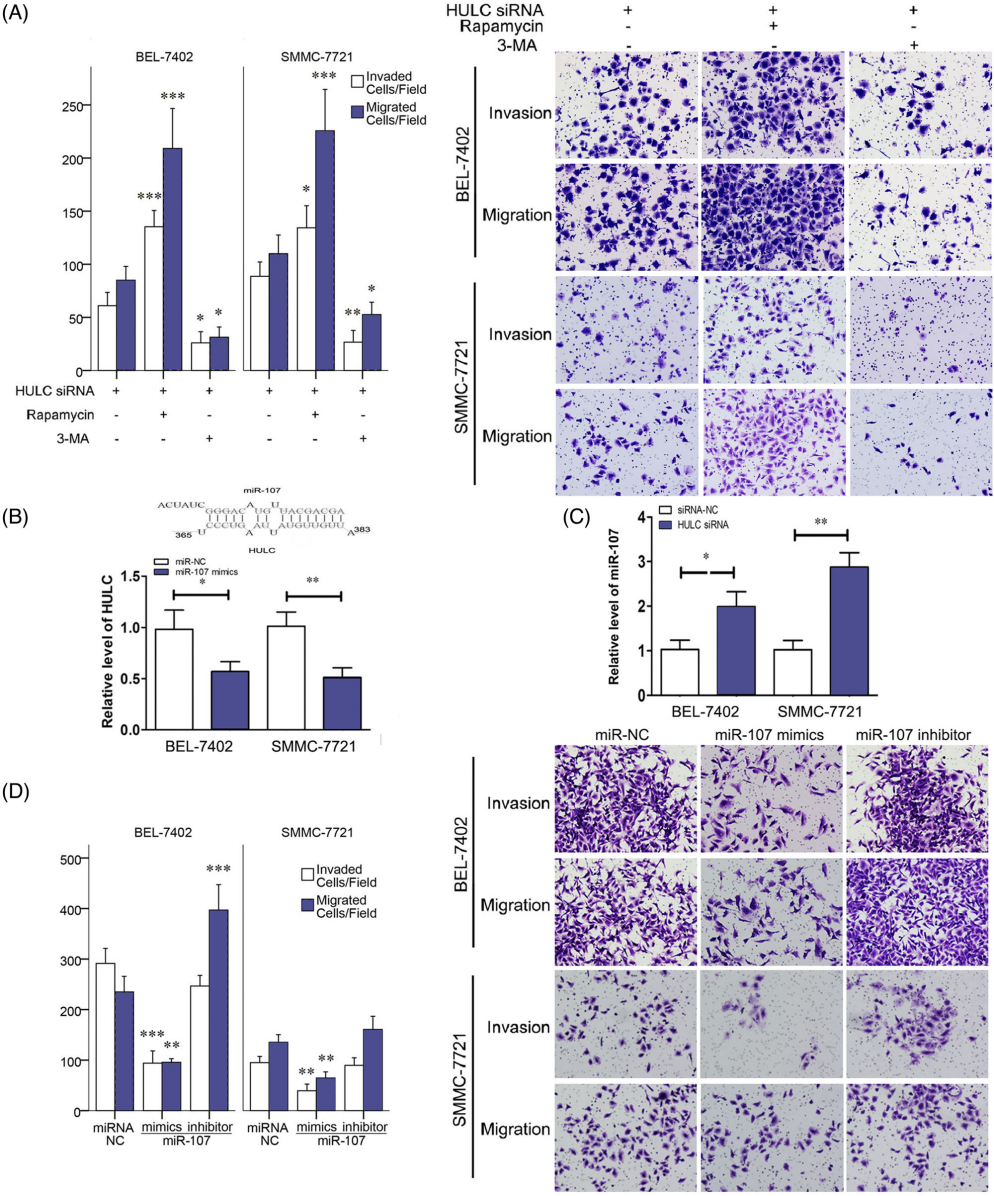
HULC‐promoted invasion via miR‐107 and autophagy. A, Invasion and migration of SMMC‐7721 and BEL‐7402 cell lines were showed in transwell assays after HULC siRNA, HULC siRNA+Rapamycin, or HULC siRNA+3‐MA treatment. B, The predicted interaction between HULC and miR‐107 through complementary base‐pairs and qRT‐PCR results of HULC in BEL‐7402 and SMMC‐7721 cells transfected with miR‐107 NC or miR‐107 mimic. C, qRT‐PCR results of miR‐107 in BEL‐7402 and SMMC‐7721 cells transfected with siRNA‐NC or HULC siRNA. D, Invasion and migration of SMMC‐7721 and BEL‐7402 cell lines in transwell assays, after miR‐107 NC, miR‐107 mimic, or miR‐107 inhibitor treatment. *P < .05, **P < .01, and ***P < .001 (compared with the first group)

**FIGURE 4:**
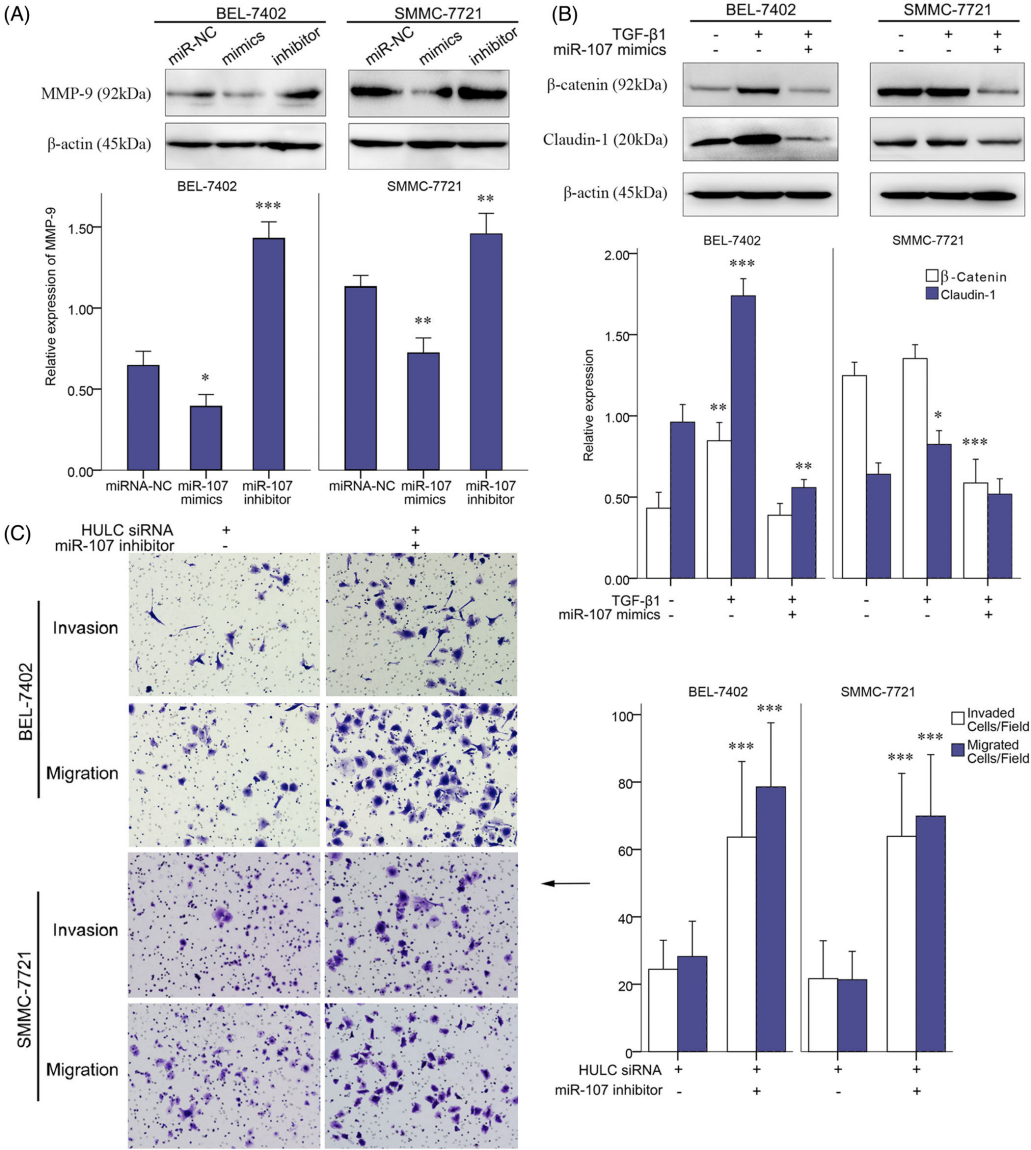
Role of miR‐107 on invasion of BEL‐7402 and SMMC‐7721 cells. A, Expressions of MMP‐9 quantified by western blots analysis in BEL‐7402 and SMMC‐7721 cells treated with miR‐107 NC, miR‐107 mimic, or miR‐107 inhibitor. B, Expressions of β‐catenin and claudin‐1 quantified by western blots analysis in the control, TGF‐β1, and TGF‐β1+miR‐107 mimic groups of BEL‐7402 and SMMC‐7721 cells. C, Invasion and migration of SMMC‐7721 and BEL‐7402 cell lines in transwell assays, after HULC siRNA or HULC siRNA+miR‐107 inhibitor treatment. *P < .05, **P < .01, and ***P < .001 (compared with the first group)

**FIGURE 6:**
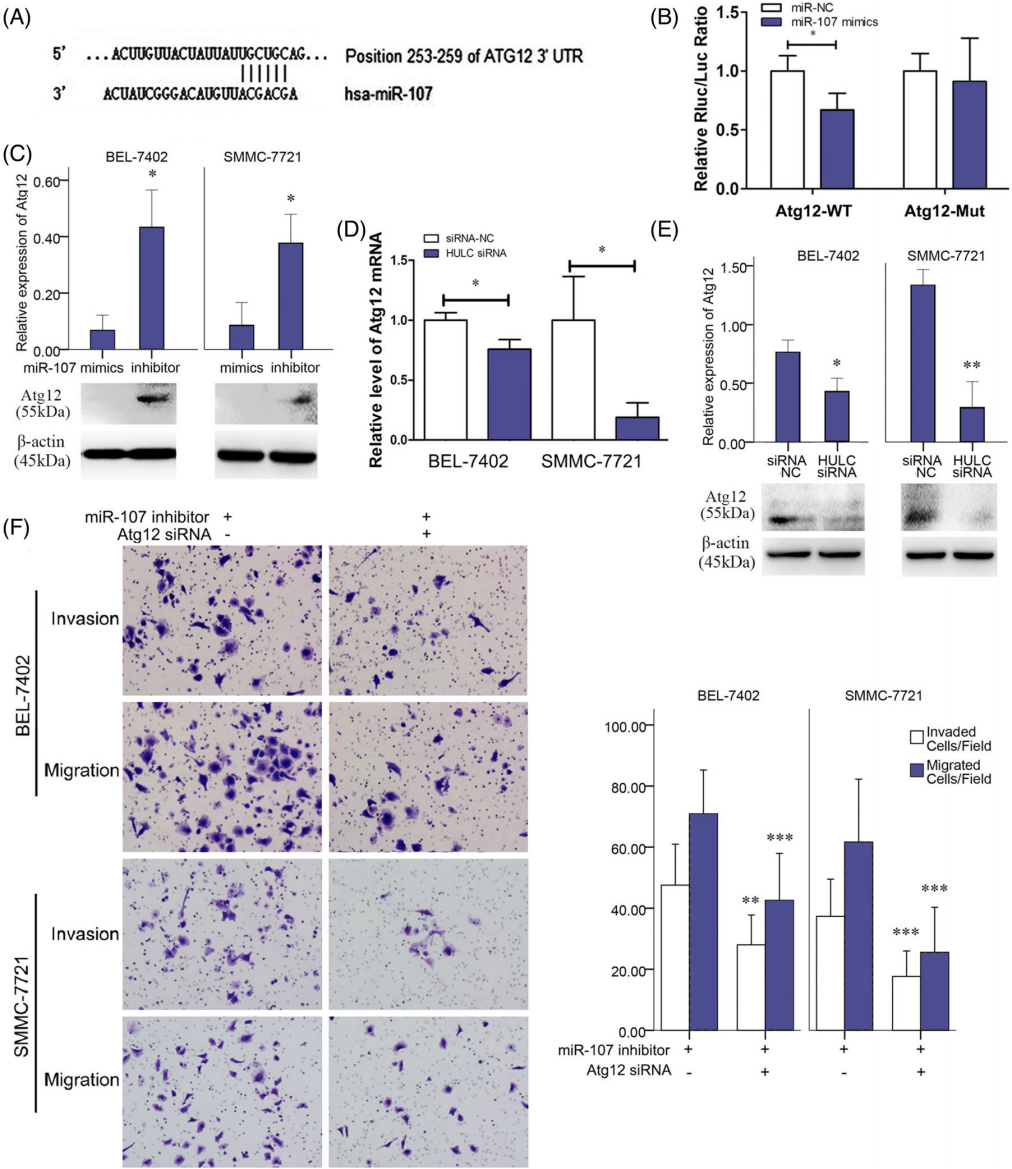
miR‐107‐inhibited autophagy through interfering with Atg12 mRNA. A, The predicted interaction between miR‐107 and Atg12 mRNA through complementary base‐pairs. B, In SMMC‐7721 and BEL‐7402 cells, luciferase activities in SMMC‐7721 cells transfected with miR‐107 NC or miR‐107 mimic, together with luciferase reporter plasmid harboring wild‐type sequence of Atg12 mRNA or mutated sequence of Atg12 mRNA. C, Expressions of Atg12 quantified by western blots analysis after miR‐107 mimic or miR‐107 inhibitor treatment. D, Expressions of Atg12 mRNA quantified by qRT‐PCR in siRNA NC and HULC siRNA groups. E, Expressions of Atg12 quantified by western blots analysis in siRNA NC and HULC siRNA groups. F, Invasion and migration of cancer cells in miR‐107 inhibitor or miR‐107 inhibitor+Atg12 siRNA group. *P < .05, **P < .01, and ***P < .001 (compared with the first group)
