# Supplementary material for: Interference of miR‐107 with Atg12 is inhibited by HULC to promote metastasis of hepatocellular carcinoma
Source: MedComm (2020). 2020 Aug 20;1(2):165–77. doi: 10.1002/mco2.25 (PMC8491224; doi:10.1002/mco2.25)
Supplement: Supplementary file 1 — Supporting Information [file MCO2-1-165-s001.docx]

Supplementary table 1 Sequences of primers

| Primer name | Sequence |
| --- | --- |
| HULC-forward | TCATGATGGAATTGGAGCCTT |
| HULC-reverse | CTCTTCCTGGCTTGCAGATTG |
| Atg12-forward | TAGAG GAACACGAACCATCC |
| Atg12-reverse | CACTGCCAAAACACTCATAGAGA |
| miR-107-forward | AGCAGCATTGTACAGGGCTATCA |
| miR-107-reverse | ATCCAGTGCAGGGTCCGACC |
| miR-107 RT primer | GTCGTATCCAGTGCGTGTCGTGGAGTCGGCAATTGCACTGGATACTGA TAG |
| U6-forward | GCTTCGGCAGCACATATACTAAAAT |
| U6-reverse | CGCTTCACGAATTTGCGTGTCAT |
| U6 RT primer | CGC TTC ACG AAT TTG CGT GTC AT |
| GAPDH-forward | GGAGCGAGATCCCTCCAAAAT |
| GAPDH-reverse | GGCTGTTGTCATACTTCTCATGG |
